# Supplementary material for: The evolution and phylodynamics of serotype A and SAT2 foot-and-mouth disease viruses in endemic regions of Africa
Source: Sci Rep. 2019 Apr 4;9:5614. doi: 10.1038/s41598-019-41995-4 (PMC6449503; doi:10.1038/s41598-019-41995-4)
Supplement: Supplementary file 1 — Supplementary material [file 41598_2019_41995_MOESM1_ESM.pdf]

Supplementary Material - The evolution and phylodynamics of serotype A and SAT2 foot-and-mouth disease viruses in endemic regions of Africa

S. Lycett[1,+], V.N. Tanya[4], M. Hall[2], D. King[3], S. Mazeri[1], V. Mioulet[3], N. Knowles[3], J. Wadsworth[3], K. Bachanek-Bankowska[3], N. Ngwa Victor[5], K.L. Morgan[6,+], and B.M.deC. Bronsvoort[1,*,+]

[1]The Roslin Institute at The Royal (Dick) School of Veterinary Studies, University of Edinburgh, Epidemiology Economics and Risk Assessment Group, Roslin, Midlothian, EH25 9RG, UK
[2]Institute of Evolutionary Biology, University of Edinburgh, Edinburgh, EH9 3JR, United Kingdom
[3]The Pirbright Institute, Ash Road, Pirbright, Woking, Surrey GU24 0NF, UK
[4]Cameroon Academy of Sciences, P.O. Box 1457 Yaoundé, Cameroon
[5]School of Veterinary Medicine and Sciences, B.P. 454, University of Ngaoundere, Ngaoundere, Cameroon
[6]Institute of Ageing and Chronic Disease and School of Veterinary Science, University of Liverpool, Leahurst Campus, Neston, Wirral, CH64 7TE, UK
[*]mark.bronsvoort@roslin.ed.ac.uk
[+]these authors contributed equally to this work

ABSTRACT

Supplementary material containing additional details on the methods used for Field Work, Laboratory analysis, and supplementary figures S1-S8.

Methods

Field work

In June and July 2012 reported outbreak herds were visited in the Adamawa and the North West Regions of Cameroon. Using an established network of contacts in the local Ministry of Agriculture (MINEPIA) and veterinarian and para-veterinarians in the field herds were identified as having or recently having FMD (or believed to have had by the owners). All herds reported to the team during the field trip were visited by the team. A standard short questionnaire was used to record recent relevant history of the herd such as movements or purchases and permission was then sought to sample ∼5 juvenile animals (<3 years old) animals per herd. The owner or herdsman was asked to select animals that he believed had lesions or had recently had signs consistent with FMD. Animals were cast in lateral recumbency and examined for clinical signs of FMD and an oropharngeal (probang) sample collected. Samples were suspended in 20ml of buffered phosphate and 1.8 ml of fluid was then drawn off with a Pasteur pipette trying to ensure plenty of cellular material and decanted to a 1.8ml cryovial and immediately stored down in a liquid nitrogen shipper[1,2].

This work was carried out with Cameroonian Ministère de l'Élevage des Pêches et Industries Animales (MINEPIA) staff, following local guidelines and regulations, as part of their disease surveillance activities and had approval from the Cameroonian Academy of Science.

Laboratory analysis of isolates collected in 2012

Virus isolation was attempted from all samples in either primary bovine thyroid cell cultures for all epithelial and oropharyngeal samples or renal swine cell cultures (RS) for epithelial and pig whole-blood samples by the WRL standard protocol[3]. All cultures showing FMDV cytopathic effect were harvested and serotyped with the WRL indirect sandwich enzyme-linked immunosorbent assay[3–5].

All viruses that were sequenced underwent a maximum of two passages in cell culture. The viral RNA was extracted from 460 μl of either the original viral suspension or the tissue culture supernatant, following the manufacturer's instructions (RNeasy Protect minikit; Qiagen Ltd.), eluted into 50 μl of diethyl pyrocarbonate-treated H_2O, and stored at $-70\,^{\circ}$C until

used for reverse transcription. A one-stage reverse transcription-PCR was used to amplify the 3' end of the VP1 gene from 5 μl of purified RNA according to the manufacturer's instructions (Ready-To-Go reverse transcription-PCR tube; Amersham Pharmacia Biotech United Kingdom Ltd.). The primers were used at a concentration of 0.2 to 0.5 pmol/μl and depended on the FMDV serotype[2]. For the reverse transcription phase, the samples were incubated at 42 °C for 30 min, followed by 5 min at 90 °C to inactivate the reverse transcriptase. After further incubation at 94 °C for 3 min, the samples were subjected to 30 cycles of PCR, with 1 cycle consisting of template denaturation at 94 °C for 1 min, primer annealing at 55 °C for 1 min, and primer extension at 72 °C for 1.5 min. There was a final incubation at 72 °C for 5 min. Amplicons were purified to remove unincorporated primers and nucleotides (Wizard Prep DNA purification kit; Promega) and extracted with phenol-chloroform to remove any viral protein or infectivity. The purified amplicons were sequenced by MWG-Biotech as recommended by the manufacturer (ABI Prism BigDye Terminators version 3.0 cycle sequencing kits; Applied Biosystems). All amplicons were forward sequenced with the PCR primer for that serotype and reverse sequenced with internal primer NK72.

A total of 19 herds were reported to the team during June/July 2012 (9 from the Adamawa and 10 from the North West Regions), and from these 87 animals were clinically examined and probang samples collected. Virus was successfully cultured and sequenced from 18/87 samples, 9 SAT2 positive animals and 9 A positive animals. The 9 animals from which SAT2 was recovered had foot lesions and 8/9 also had mouth lesions. Similarly 8/9 animals from which serotype A was recovered had foot lesions and 7/9 had mouth lesions. Of those for which no virus was cultured 59/69 had foot lesions and 52/69 had mouth lesions. The sample codes and accession numbers of the sequenced samples are given in Table 1 in the main paper.

In order to relate the new sequences to previously circulating strains, further A and SAT2 VP1 sequences were accessed from our previous study[2] and combined with other publicly available sequences from Genbank (http://www.ncbi.nlm.nih.gov/genbank/) as well as recent sequences provided by Ludi et al.[6]. In total there were sequences available for Cameroon from 2000, 2005, 2012, 2013 and 2014.

References

1. Kitching, R. P. & Donaldson, A. I. Collection and transportation of specimens for vesicular virus investigation. *Revue Sci. et Tech. de l'Office Int. des Epizoot.* **6**, 263–272 (1987).

2. Bronsvoort, B., Radford, A., Tanya, V. N., Kitching, R. P. & Morgan, K. L. The molecular epidemiology of foot-and-mouth disease viruses in the Adamawa Province of Cameroon. *J. Clin. Microbiol.* **42**, 2186–2196 (2004).

3. Anon. Chapter 2.1.1, Foot and Mouth Disease. In *Manual of Diagnostic Tests and Vaccines for Terrestrial Animals*, vol. 2005 (O.I.E., 2005), 5th edn.

4. Ferris, N. P., Powell, H. & Donaldson, A. I. Use of Pre-Coated Immunoplates and Freeze-Dried Reagents For the Diagnosis of Foot-and-Mouth-Disease and Swine Vesicular Disease By Enzyme-Linked Immunosorbent-Assay (Elisa). *J. Virol. Methods* **19**, 197–206 (1988).

5. Roeder, P. L. & Le Blanc Smith, P. M. Detection and typing of foot-and-mouth disease virus by enzyme-linked immunosorbent assay: a sensitive, rapid and reliable technique for primary diagnosis. *Res. veterinary science* **43**, 225–32 (1987).

6. Ludi, A. *et al.* Serotype Diversity of Foot-and-Mouth-Disease Virus in Livestock without History of Vaccination in the Far North Region of Cameroon. *Transboundary Emerg. Dis.* 1–12 (2014). DOI 10.1111/tbed.12227.

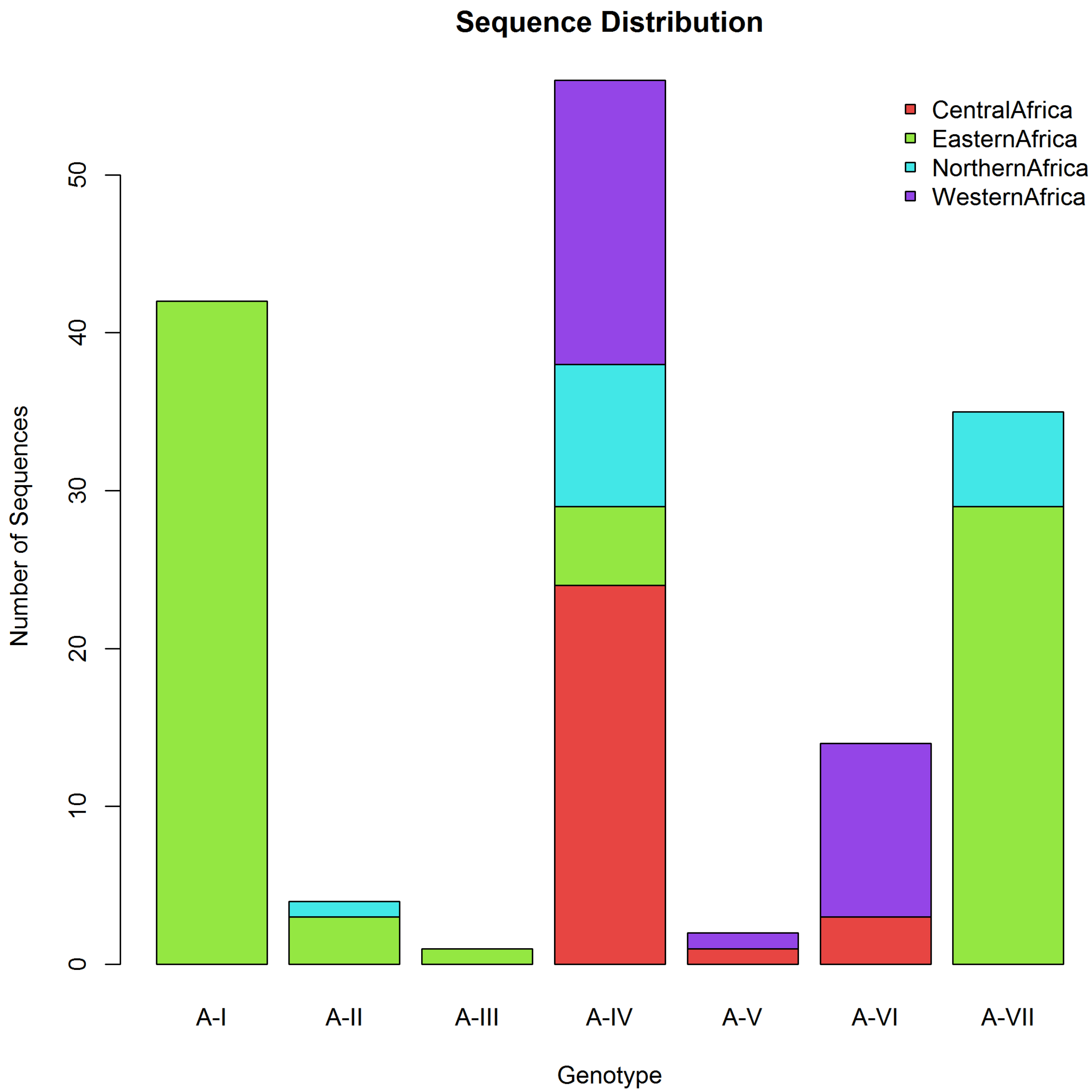

Figure S1. Composition of 154 serotype A sequence dataset by genotype and region.

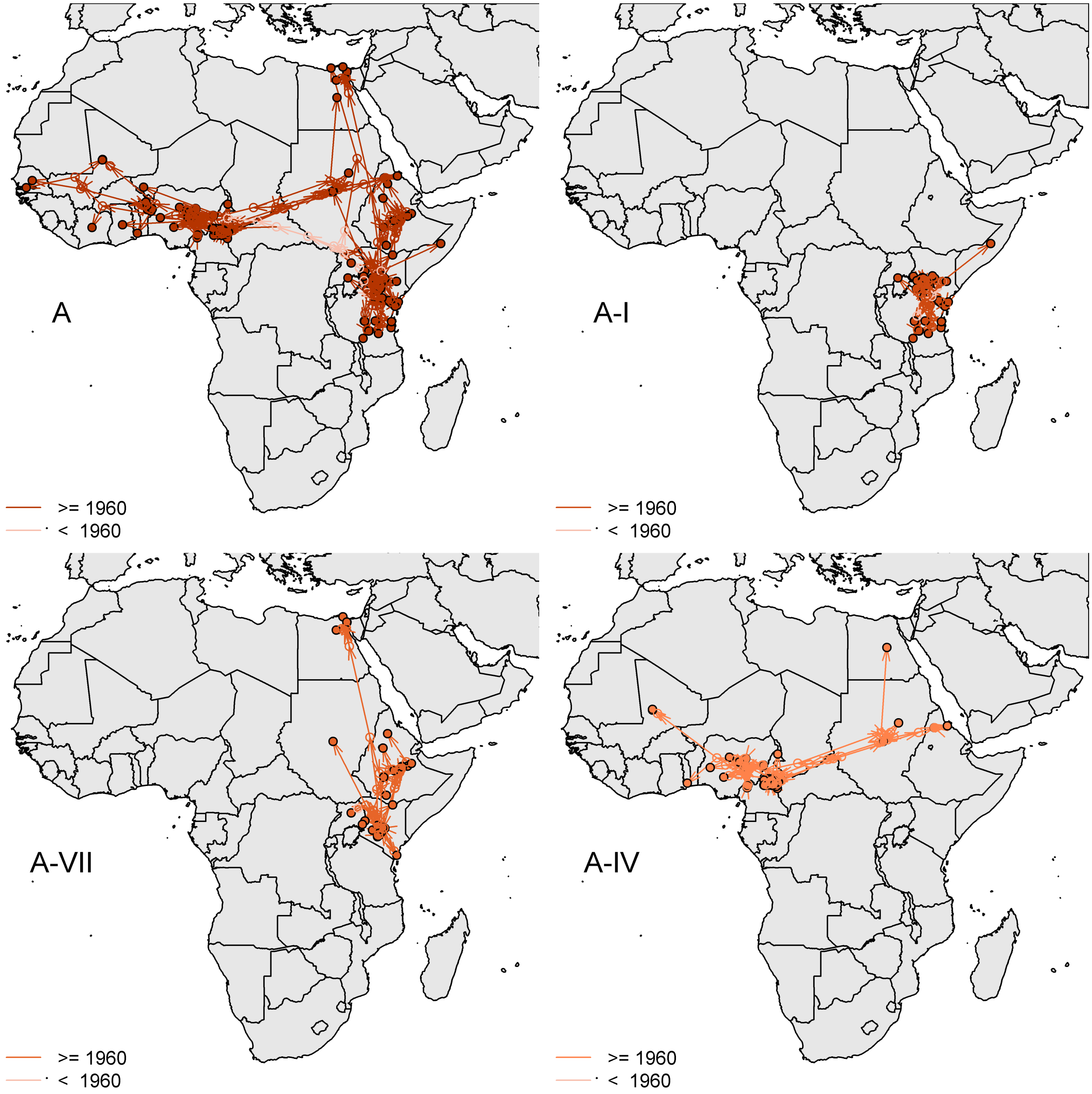

Figure S2. Spatial projection of maximum clade credibility trees from serotype A sequences. Spatial components were inferred using homogenous Brownian motion and projected onto maps. Upper left: MCC tree from all serotype A dataset. Upper right: MCC tree from A-I sequences only, Lower left: A-VII, Lower right: A-IV. Filled circles are the positions of the sequences, open circles are the inferred positions of the ancestral nodes. Dark arrows indicate tree branches and inferred transmission routes with dates $\geq$ 1960, pale arrows indicate older routes (< 1960).

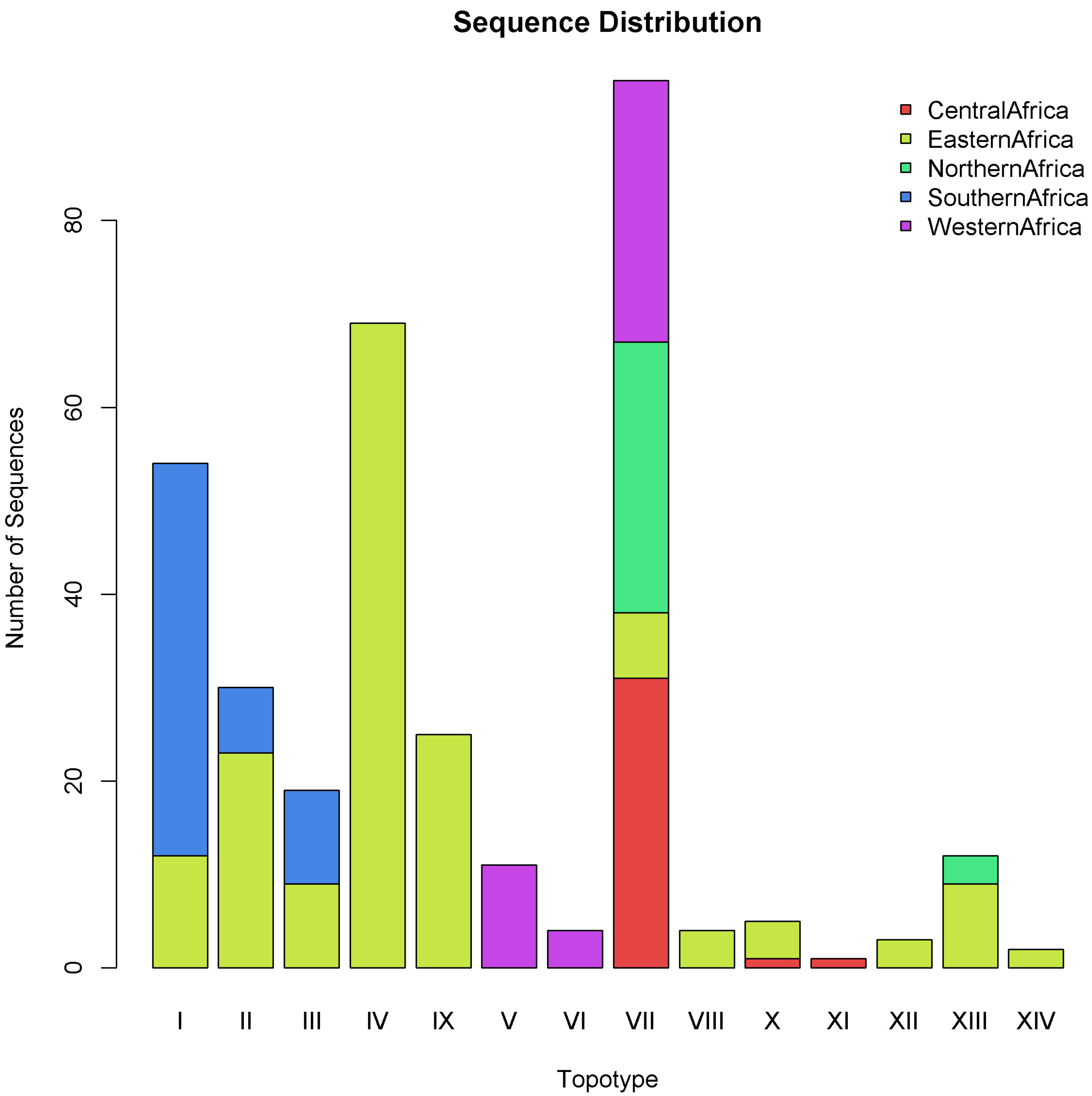

Figure S3. Composition of 334 serotype SAT2 sequence data by topotype and region.

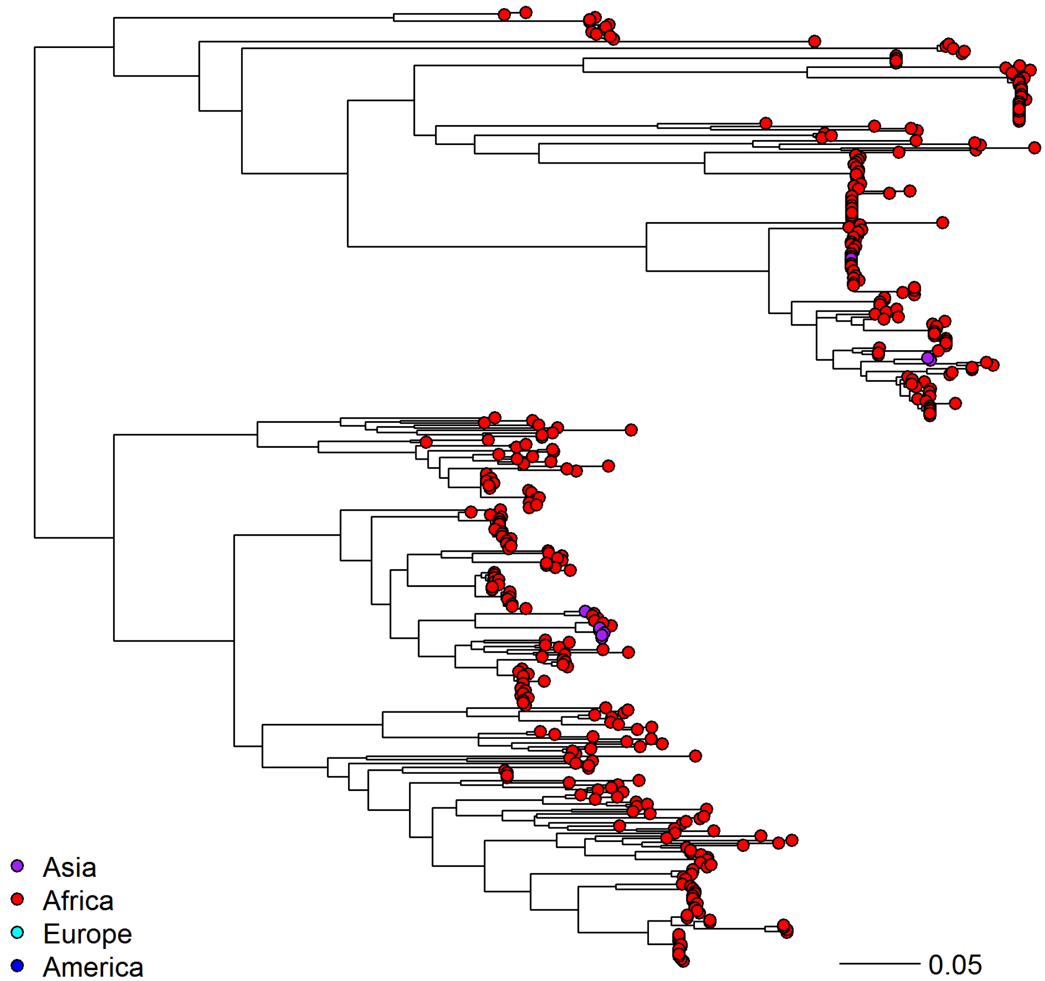

Figure S4. Maximum likelihood tree from world wide SAT2 sequences, showing the majority of sequences are from Africa, with only occasional incursions into Asia.

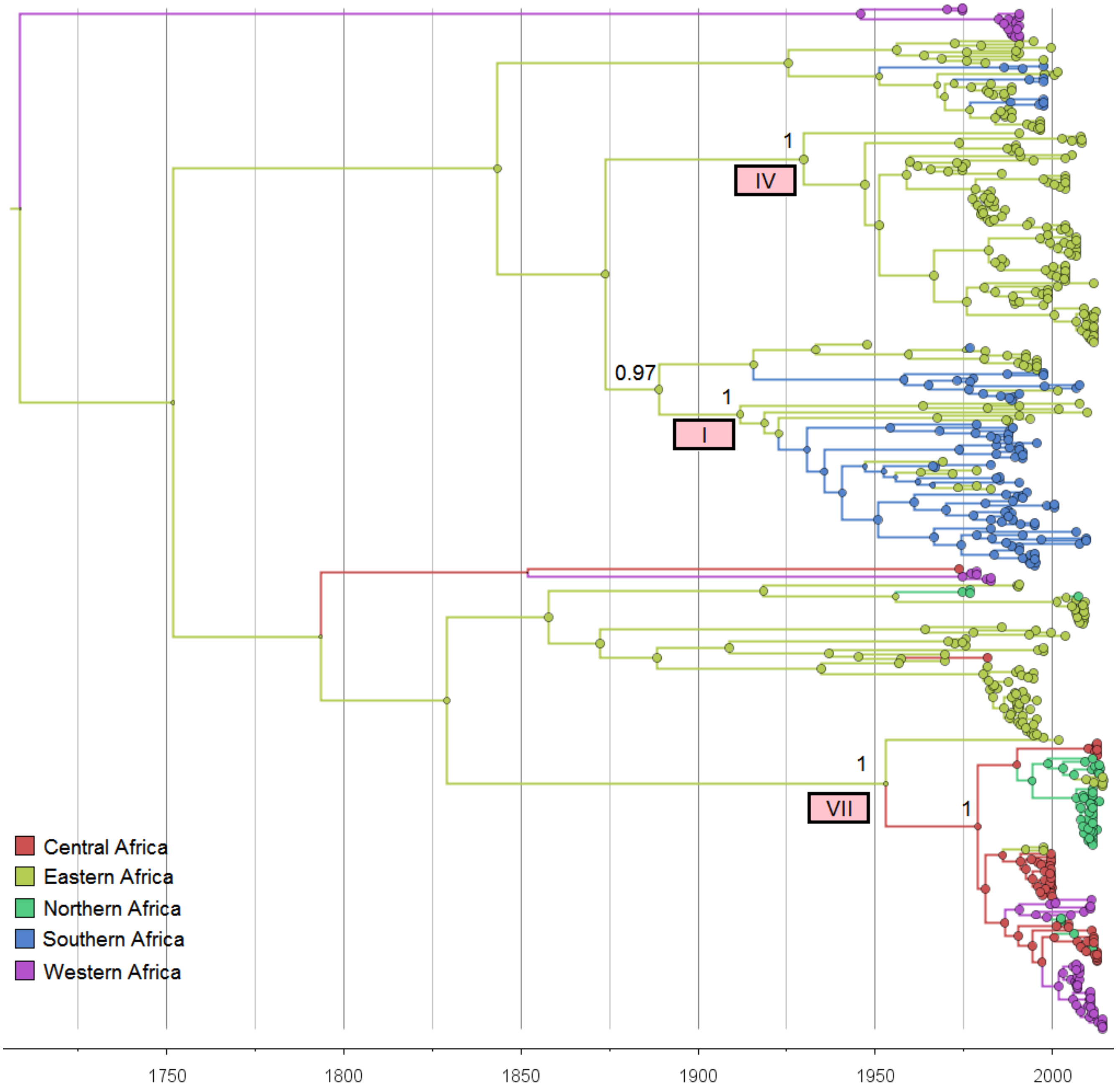

Figure S5. **Time scaled tree of 334 SAT2 sequences from Africa**, with genotypes used in further analysis marked.

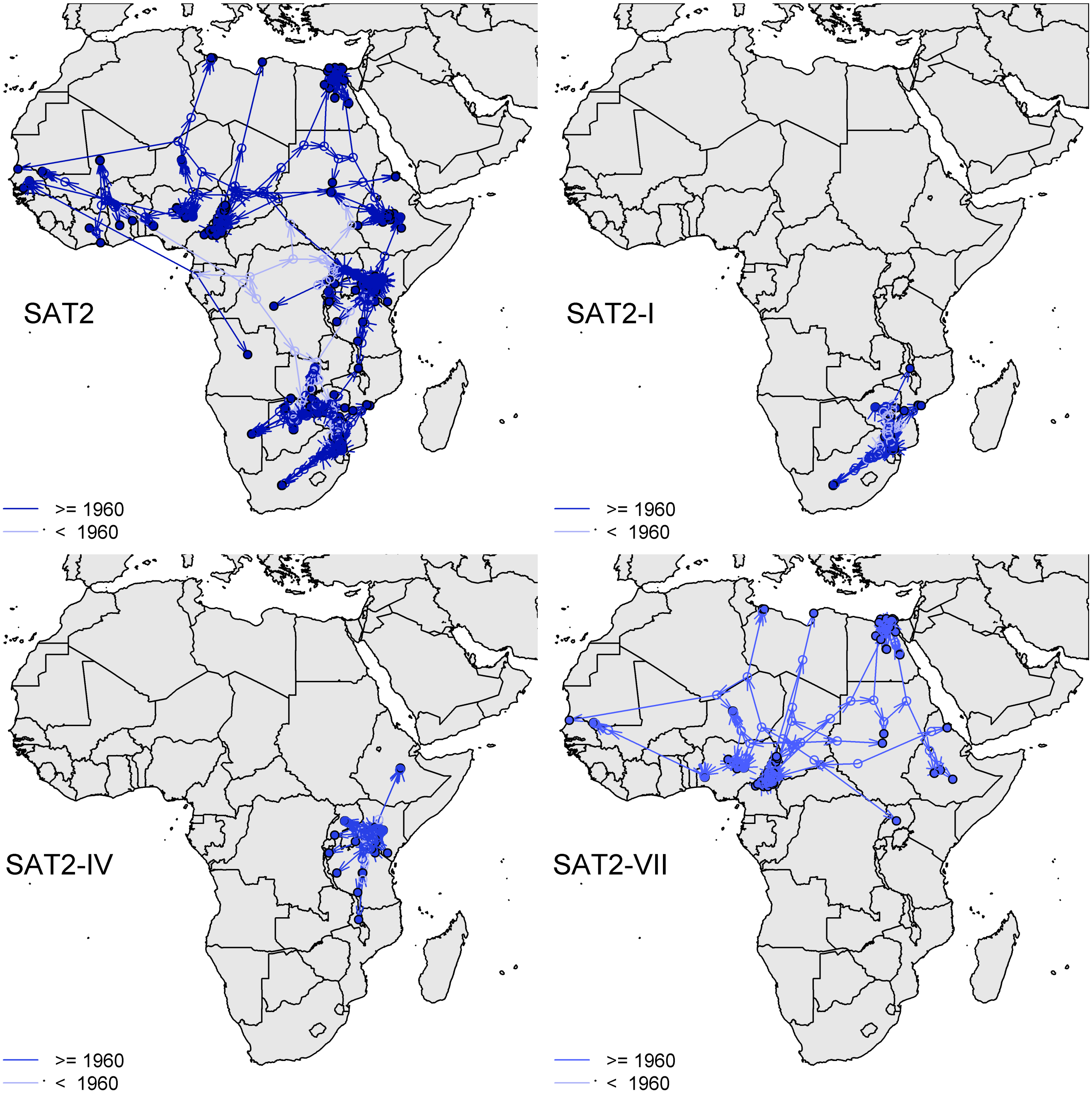

Figure S6. Spatial projection of maximum clade credibility trees from serotype SAT2 sequences. Spatial components were inferred using homogenous Brownian motion and projected onto maps. Upper left: MCC tree from all serotype SAT2 dataset. Upper right: MCC tree from Topotype SAT2-I sequences only, Lower left: SAT2-IV, Lower right: SAT2-VII. Filled circles are the positions of the sequences, open circles are the inferred positions of the ancestral nodes. Dark arrows indicate tree branches and inferred transmission routes with dates $\geq$ 1960, pale arrows indicate older routes ($<$ 1960).

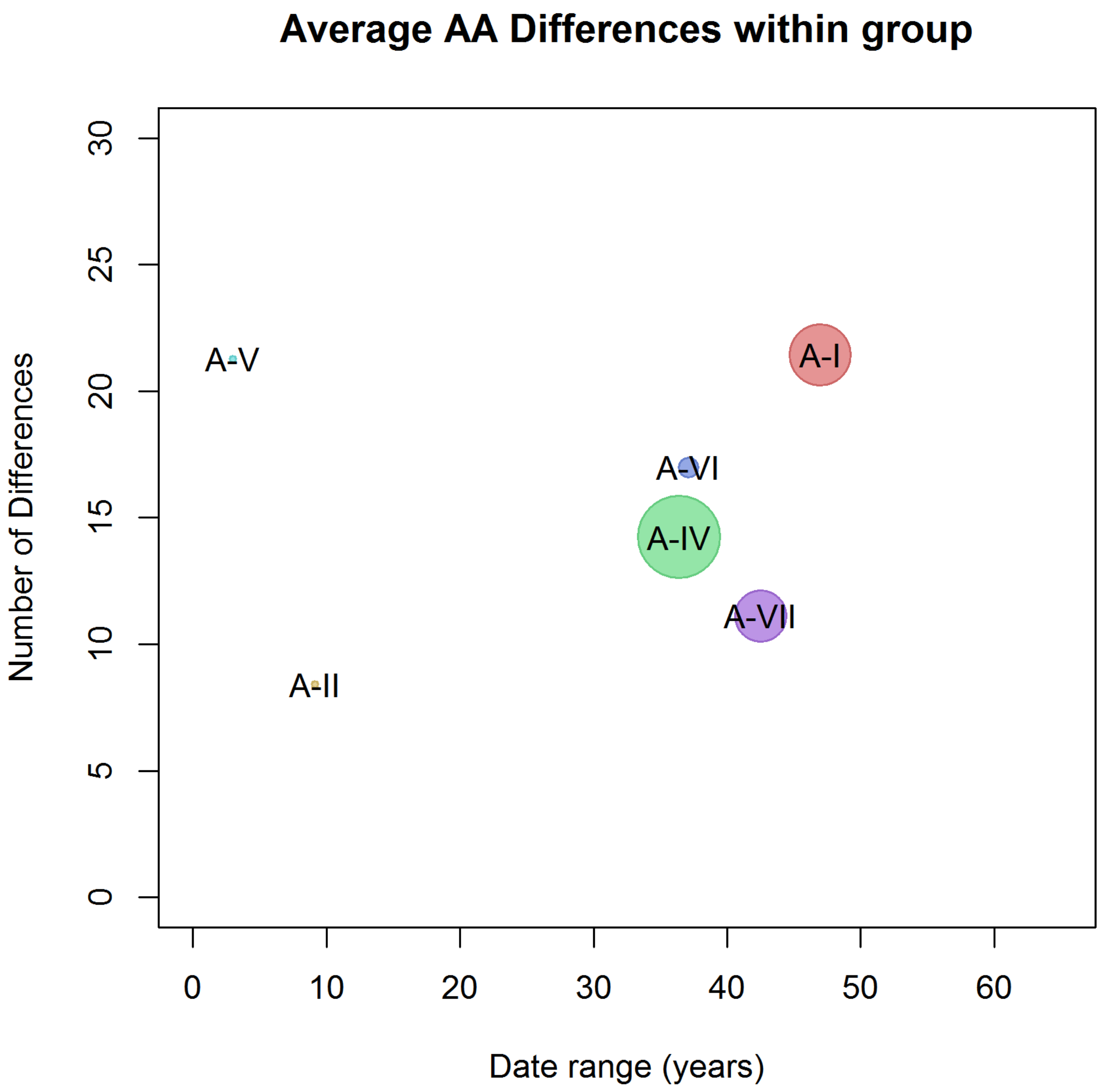

Figure S7. Average number of pairwise amino acid differences for serotype A within A groups as a function of the date range of the sequences (youngest – oldest). Circle size indicates the number of sequences within each group (see Figure S1).

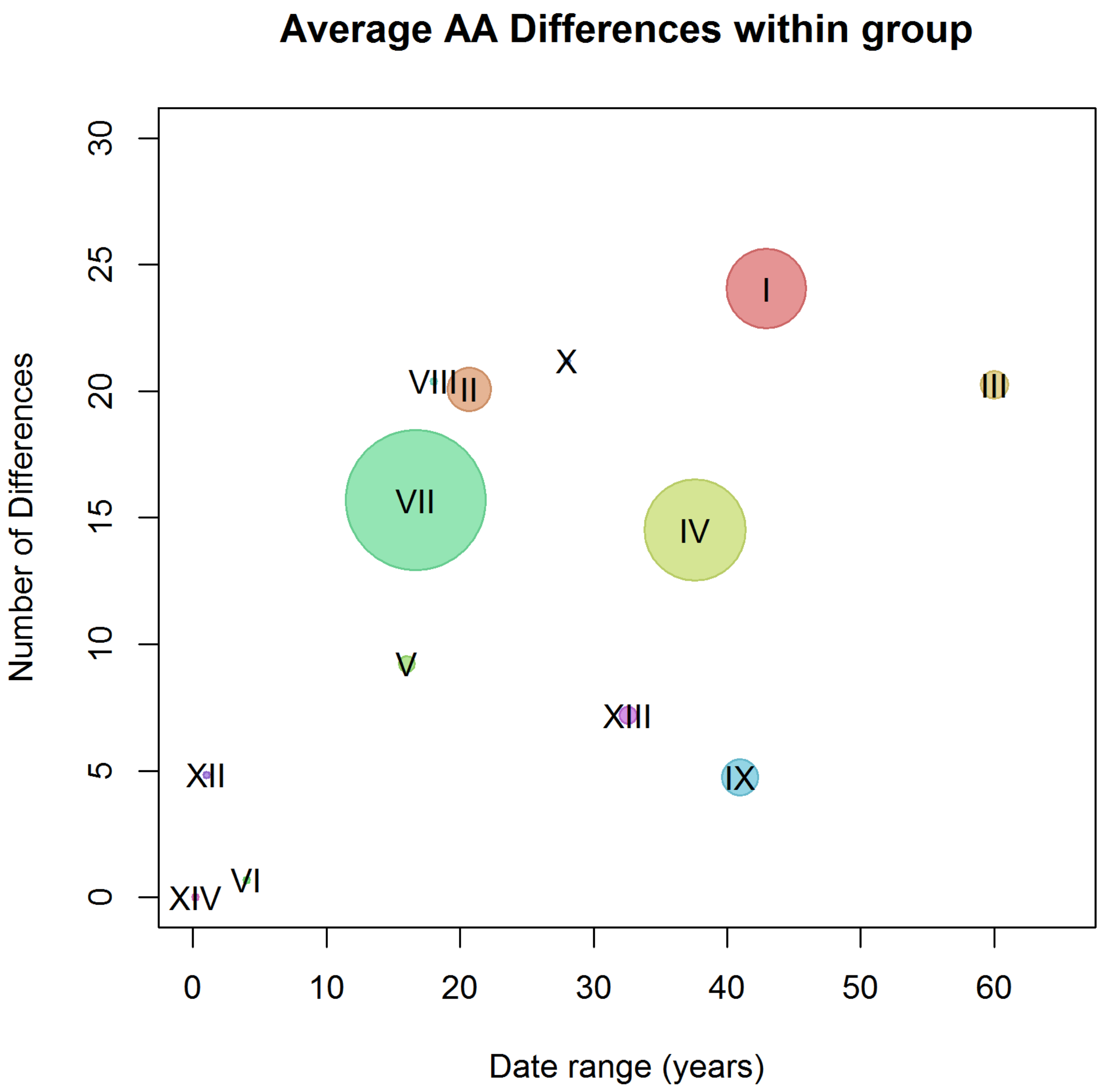

Figure S8. Average number of pairwise amino acid differences for serotype SAT2 within Topotypes as a function of the date range of the sequences (youngest – oldest). Circle size indicates the number of sequences within each group (see Figure S2).

A and SAT2 within and between group amino acid variations

Within group stats for A

Group (A)	NumSeqs	Mean	Median	StDev	MinDate	MaxDate
(ALL)	154	0.112	0.118	0.034	1964.500	2013.888
A-I	42	0.096	0.103	0.040	1966.500	2013.500
A-II	4	0.038	0.038	0.034	1972.362	1981.500
A-III	1	NA	NA	NA	1964.500	1964.500
A-IV	56	0.063	0.066	0.023	1977.500	2013.888
A-V	2	0.095	0.095	NA	1973.500	1976.500
A-VI	14	0.076	0.085	0.032	1973.500	2010.608
A-VII	35	0.050	0.052	0.017	1966.096	2008.581

Between Group Stats for A

Group	A-I	A-II	A-III	A-IV	A-V	A-VI	A-VII
A-I	0.096	0.135	0.122	0.130	0.121	0.121	0.128
A-II	0.135	0.038	0.123	0.134	0.118	0.125	0.130
A-III	0.122	0.123	NA	0.114	0.100	0.120	0.116
A-IV	0.130	0.134	0.114	0.063	0.114	0.121	0.131
A-V	0.121	0.118	0.100	0.114	0.095	0.097	0.113
A-VI	0.121	0.125	0.120	0.121	0.097	0.076	0.107
A-VII	0.128	0.130	0.116	0.131	0.113	0.107	0.050

No. Sites 223

Between groups in number of AA changes for A

Group	A-I	A-II	A-III	A-IV	A-V	A-VI	A-VII
A-I	21	30	27	29	27	27	28
A-II	30	8	27	30	26	28	29
A-III	27	27	NA	25	22	27	26
A-IV	29	30	25	14	25	27	29
A-V	27	26	22	25	21	22	25
A-VI	27	28	27	27	22	17	24
A-VII	28	29	26	29	25	24	11

Within Topotype Stats for SAT2

TopoType	NumSeqs	Mean	Median	StDev	MinDate	MaxDate
(ALL)	334	0.162	0.176	0.049	1948.500	2015.189
I	54	0.107	0.108	0.028	1967.770	2010.701
II	30	0.090	0.094	0.041	1981.847	2002.500
III	19	0.090	0.097	0.033	1948.500	2008.500
IV	69	0.065	0.069	0.024	1975.500	2013.104
V	11	0.041	0.028	0.034	1975.500	1991.500
VI	4	0.003	0.005	0.002	1979.500	1983.500
VII	95	0.070	0.074	0.029	1998.500	2015.189
VIII	4	0.091	0.097	0.027	1986.500	2004.500
IX	25	0.021	0.005	0.035	1957.500	1998.500
X	5	0.095	0.102	0.029	1970.500	1998.500
XI	1	NA	NA	NA	1974.500	1974.500
XII	3	0.022	0.023	0.003	1975.500	1976.500
XIII	12	0.032	0.009	0.040	1977.500	2010.066
XIV	2	0.000	0.000	NA	1991.329	1991.500

Between Topotype Stats for SAT2

TopoType	I	II	III	IV	V	VI	VII	VIII	IX	X	XI	XII	XIII	XIV
I	0.107	0.167	0.143	0.141	0.152	0.179	0.186	0.168	0.180	0.179	0.217	0.187	0.186	0.163
II	0.167	0.090	0.167	0.176	0.178	0.196	0.223	0.200	0.195	0.213	0.201	0.220	0.205	0.192
III	0.143	0.167	0.090	0.135	0.163	0.188	0.199	0.183	0.165	0.193	0.212	0.207	0.191	0.170
IV	0.141	0.176	0.135	0.065	0.173	0.191	0.188	0.172	0.185	0.192	0.212	0.201	0.190	0.178
V	0.152	0.178	0.163	0.173	0.041	0.166	0.186	0.142	0.145	0.159	0.208	0.169	0.165	0.151
VI	0.179	0.196	0.188	0.191	0.166	0.003	0.234	0.208	0.204	0.210	0.222	0.225	0.224	0.201
VII	0.186	0.223	0.199	0.188	0.186	0.234	0.070	0.171	0.181	0.177	0.237	0.201	0.177	0.174
VIII	0.168	0.200	0.183	0.172	0.142	0.208	0.171	0.091	0.141	0.139	0.218	0.142	0.151	0.159
IX	0.180	0.195	0.165	0.185	0.145	0.204	0.181	0.141	0.021	0.128	0.217	0.154	0.163	0.127
X	0.179	0.213	0.193	0.192	0.159	0.210	0.177	0.139	0.128	0.095	0.235	0.143	0.175	0.147
XI	0.217	0.201	0.212	0.212	0.208	0.222	0.237	0.218	0.217	0.235	NA	0.247	0.221	0.222
XII	0.187	0.220	0.207	0.201	0.169	0.225	0.201	0.142	0.154	0.143	0.247	0.022	0.188	0.165
XIII	0.186	0.205	0.191	0.190	0.165	0.224	0.177	0.151	0.163	0.175	0.221	0.188	0.032	0.149
XIV	0.163	0.192	0.170	0.178	0.151	0.201	0.174	0.159	0.127	0.147	0.222	0.165	0.149	0.000

No. Sites 224

Between topotype in number of AA changes for SAT2

TopoType	I	II	III	IV	V	VI	VII	VIII	IX	X	XI	XII	XIII	XIV
I	24	37	32	32	34	40	42	38	40	40	49	42	42	36
II	37	20	37	39	40	44	50	45	44	48	45	49	46	43
III	32	37	20	30	36	42	45	41	37	43	47	46	43	38
IV	32	39	30	15	39	43	42	39	41	43	48	45	43	40
V	34	40	36	39	9	37	42	32	33	36	46	38	37	34
VI	40	44	42	43	37	1	52	47	46	47	50	50	50	45
VII	42	50	45	42	42	52	16	38	41	40	53	45	40	39
VIII	38	45	41	39	32	47	38	20	32	31	49	32	34	36
IX	40	44	37	41	33	46	41	32	5	29	49	34	36	29
X	40	48	43	43	36	47	40	31	29	21	53	32	39	33
XI	49	45	47	48	46	50	53	49	49	53	NA	55	50	50
XII	42	49	46	45	38	50	45	32	34	32	55	5	42	37
XIII	42	46	43	43	37	50	40	34	36	39	50	42	7	33
XIV	36	43	38	40	34	45	39	36	29	33	50	37	33	0

Figure S9. Table of within and between group amino acid differences for A and SAT2

Comparison of A and SAT2 clade diversities

Clock Rate	A	A-I	A-VII	A-IV	SAT2	SAT2-IV	SAT2-I	SAT2-VII
Mean	0.00566	0.00431	0.00401	0.00865	0.00320	0.00066	0.00302	0.00743
Median	0.00563	0.00407	0.00387	0.00857	0.00318	0.00065	0.00291	0.00727
Q1	0.00519	0.00321	0.00321	0.00805	0.00289	0.00058	0.00232	0.00651
Q3	0.00610	0.00517	0.00459	0.00919	0.00348	0.00072	0.00357	0.00820
Lower	0.00408	0.00141	0.00153	0.00638	0.00206	0.00037	0.00104	0.00443
Upper	0.00744	0.00793	0.00663	0.01090	0.00437	0.00093	0.00542	0.01071
Lower 95% HPD	0.00447	0.00159	0.00223	0.00711	0.00240	0.00045	0.00148	0.00501
Upper 95% HPD	0.00685	0.00736	0.00642	0.01053	0.00407	0.00090	0.00475	0.01013

Root Age	A	A-I	A-VII	A-IV	SAT2	SAT2-IV	SAT2-I	SAT2-VII
Mean	1936.4	1921.6	1955.1	1974.9	1693.0	1752.0	1900.0	1977.3
Median	1937.3	1926.4	1956.7	1974.9	1709.1	1759.9	1905.2	1982.7
Q1	1932.6	1908.8	1951.5	1974.0	1655.3	1723.2	1883.5	1972.8
Q3	1941.5	1939.3	1960.4	1975.8	1752.2	1790.1	1919.0	1988.5
Lower	1919.2	1863.0	1938.2	1971.5	1510.1	1624.1	1830.3	1949.3
Upper	1953.6	1960.7	1966.1	1977.5	1867.0	1881.1	1952.9	1995.7
Lower 95% HPD	1922.1	1875.2	1940.0	1972.7	1496.8	1626.9	1849.1	1944.4
Upper 95% HPD	1949.7	1955.5	1966.1	1977.5	1810.3	1846.3	1943.2	1995.1

Number of AA Differences	A	A-I	A-VII	A-IV	SAT2	SAT2-IV	SAT2-I	SAT2-VII
Mean	25.0	21.4	11.1	14.2	36.3	14.5	24.0	15.7
Median	26.4	23.1	11.6	14.7	39.4	15.6	24.2	16.6
Q1	22.1	17.8	9.5	11.3	32.1	12.4	20.5	12.4
Q3	30.5	28.4	13.7	17.9	43.6	17.7	28.0	19.0
Lower	9.5	2.1	3.2	2.1	15.1	5.2	9.4	3.1
Upper	41.3	37.9	20.0	25.0	59.7	25.4	39.0	28.7
Lower 95% HPD	8.4	2.1	3.1	5.0	13.1	1.0	11.4	1.0
Upper 95% HPD	36.3	35.8	18.0	23.8	54.4	22.1	35.9	28.0

Diffusion rate	A	A-I	A-VII	A-IV	SAT2	SAT2-IV	SAT2-I	SAT2-VII
Mean	101.8	33.7	110.4	209.0	46.1	8.8	20.3	327.6
Median	101.3	32.7	108.3	205.9	45.2	8.6	19.4	322.8
Q1	94.7	25.8	95.0	190.5	41.0	7.7	16.0	286.0
Q3	108.7	41.3	125.3	221.7	49.8	9.6	23.8	371.8
Lower	73.8	9.8	51.3	146.8	28.3	5.0	6.5	160.3
Upper	129.4	63.7	167.0	267.0	62.7	12.5	35.2	500.1
Lower 95% HPD	81.1	14.7	72.2	166.1	32.5	6.0	10.3	200.7
Upper 95% HPD	123.2	52.4	153.6	260.7	60.2	12.0	32.7	451.1

Figure S10. Comparison of clock rate, root age (TMRCA), number of amino acid differences and diffusion rate for A and

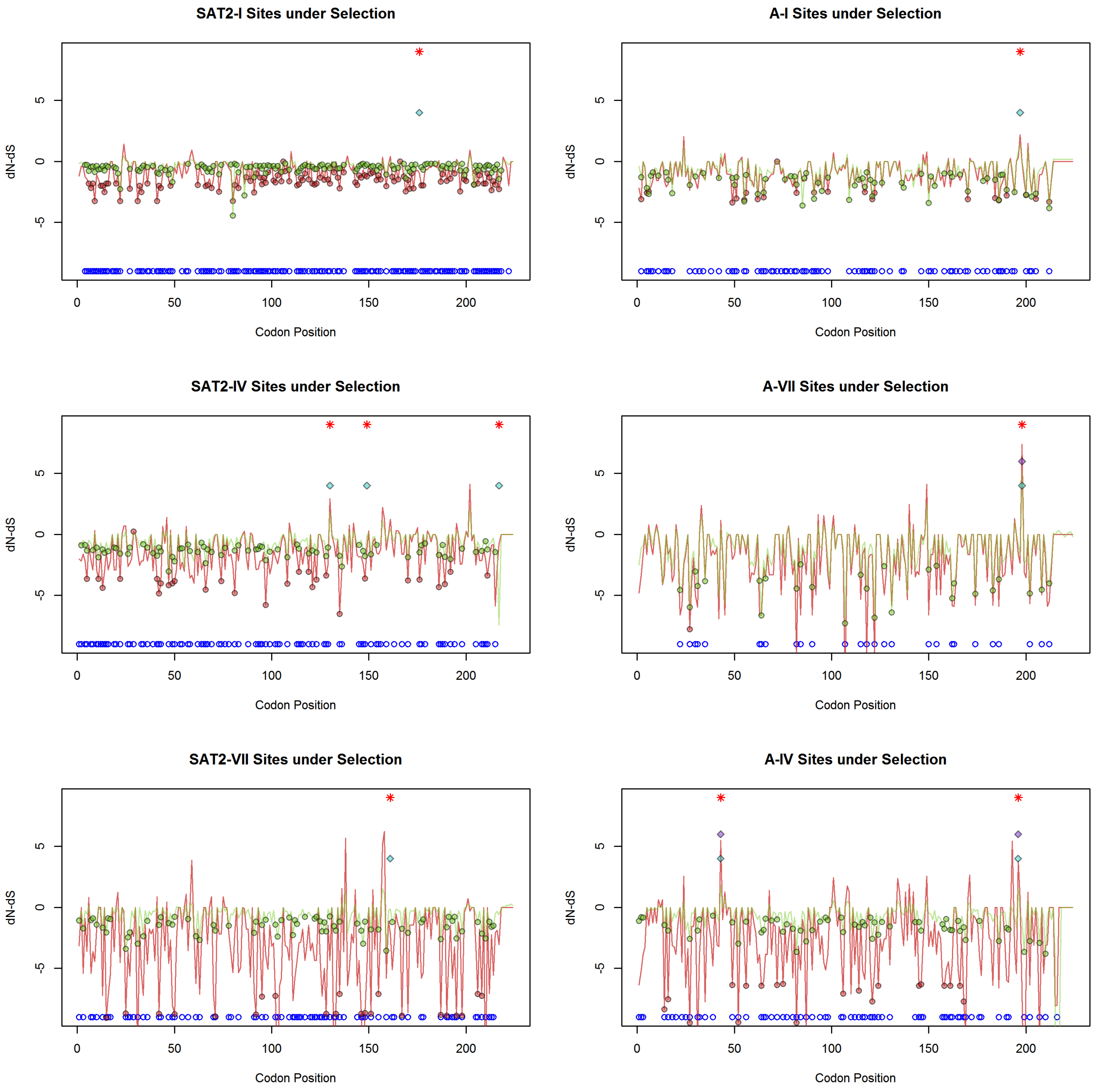

Figure S11. Sites under positive and negative selection from SLAC (red), FEL (green), MEME (blue) and FUBAR (purple). The normalised dN-dS values for SLAC and FEL are plotted, and significantly selected sites (p-value ≤ 0.01) are indicated with a circle. The sites under selection using MEME (p-value ≤ 0.01) and FUBAR sites (probability ≥ 0.99) are indicated with blue and purple diamonds respectively. Positively selected sites, significant under at least one test are indicated with a red star, and significant negatively selected sites are indicated with a blue circle.
